# Perceptions of clinical years’ medical students and interns towards assessment methods used in King Abdulaziz University, Jeddah

**DOI:** 10.12669/pjms.314.7378

**Published:** 2015

**Authors:** Nahla Khamis Ibrahim, Budoor Mohammed Al-Sharabi, Rasha Abdullah Al-Asiri, Najat Abdullah Alotaibi, Wejdan Ibrahim Al-Husaini, Hussa Adel Al-Khajah, Reem Mohammad Rakkah, Afnan Mohammed Turkistani

**Affiliations:** 1 Nahla Khamis Ibrahim, MBBCh, MPH, Dr.PH (Epidemiology), DHPE, JMHPE (Med Educ.) Professor of Epidemiology & Public Health, Family & Community Medicine Department, King Abdulaziz University, Jeddah, Saudi Arabia Epidemiology Department, High Institute of Public Health, Alexandria University, Egypt; 2 Budoor Mohammed Al-Sharabi, Intern at King Abdulaziz University, Jeddah, Saudi Arabia; 3 Rasha Abdullah Al-Asiri, Intern at King Abdulaziz University, Jeddah, Saudi Arabia; 4 Najat Abdullah Alotaibi, Intern at King Abdulaziz University, Jeddah, Saudi Arabia; 5 Wejdan Ibrahim Al-Husaini, Intern at King Abdulaziz University, Jeddah, Saudi Arabia; 6 Hussa Adel Al-Khajah, Intern at King Abdulaziz University, Jeddah, Saudi Arabia; 7 Reem Mohammad Rakkah, Intern at King Abdulaziz University, Jeddah, Saudi Arabia; 8 Afnan Mohammed Turkistani, Intern at King Abdulaziz University, Jeddah, Saudi Arabia

**Keywords:** Assessment, Medical education, Professionalism, OSCE, OSPE, Peer assessment, Faculty development

## Abstract

**Objectives:**

The study was done to determine the perception of clinical years’ medical students and interns about assessment methods used in Faculty of Medicine, King Abdulaziz University, Jeddah, Saudi Arabia.

**Methods:**

A cross sectional study was conducted during the educational year 2012/2013. A multistage stratified random sample method was used to select 600 senior medical students (4^th^-6^th^) and interns. Perception of medical students and interns about different assessment formats was inquired using 3 points Likert scale.

**Results:**

About two-fifths of participants agreed that assessment methods are comprehensive, reflecting what they taught, and challenging them. MCQs were the commonest (56.8%) preferred written assessment format. OSCE (74.1%) and OSPE (70.6%) were seen as good tools for assessing clinical competencies. Students had good perceptions towards peer assessment, log-book and open book exams. Males preferred peer assessment method more than females, with a statistical significant difference (χ^2^ = 6.43, *p*< 0.05).

**Conclusion:**

Assessment plan needs further improvements and should be designed prospectively along with learning outcomes, as only about 40 % of participants agreed with assessment items. The current development of the faculty Assessment Unit will provide much help. This will lead to better preparation of medical students for their future responsibility as tomorrow’s doctors.

## INTRODUCTION

Medical education is the art and science of medical learning which has rapidly progressed in the recent years. Adequately measuring core competencies of the medical students is a vital constituent for evaluation, provision of reliable feedback and improving medical education.^1^ Assessment is an essential compenent in medical education because of its implications on the students, teachers, communities and the whole world; after graduation from their medical schools.^2^ Assessment is an integral component of overall educational activities and a vital tool which drives student learning, because students usually tend to concentrate on the material to be assessed.^3^ Furthermore, the selected assessment method should be consistent with the curriculum defined objectives. The test contents should be carefully planned along with the learning objectives in blueprinting process^4^ and should be purpose driven.^5^

The use of a variety of assessment methods has become a characteristic of medical education, credentialing, and licensure since the 1950s.^6^ However, the evaluation of competences using traditional examination has its limitations because of low validity and reliability.^7^ Relatively new non-traditional assessment methods (log book, open-book exam, simulations, self and peer assessments and other innovative formats) have been introduced in medical education in the last decades.^8^ They are believed to be fairer as they measure qualities, skills and competences. These methods would be valuable in contexts other than the immediate context of assessment.^6^,^8^

The written traditional methods of assessment include the Multiple Choice Questions (MCQs), short and long assays, etc. Basically, MCQs exams assess the factual knowledge, recall, understanding and interpretation.^2^ On the other hand, for assessing clinical skills, Objective Structured Clinical Examination (OSCE), Objective Structured Practical Examination (OSPE), short, long cases, Mini-Clinical Evaluation Exercise (Mini-CEX), peer assessment, and other tests can be used.^9^,^10^

Clinical presentation models are designed to ensure that the medical students not only acquire appropriate scientific and clinical knowledge, but also the practical procedures and communication skills. This leads to acquiring most of the learning domains mainly cognitive, affective and psychomotor.^11^,^12^

Many researchers have been tried to identify the best alternative method for assessing medical teaching, but none has come with a clear cut answer, as different levels of knowledge and skill domains are better assessed by different types of assessment methods.^11^ Students’ perception about their assessments can be used in the process of improving quality of assessment, educational programs, student learning and performance. 13

In 2007, the Faculty of Medicine, King Abdulaziz University (KAU), Jeddah, launched a new hybrid system-based curriculum.^14^ Although evaluation of perception of medical students regarding their assessment has an essential impact on the educational process, however such evaluations are lacking,^8^ especially after the implementation of the new curriculum. So, conduction of such study on the perceptions of students towards assessments is urgently needed.

The aim of the current study was to determine the perception of senior medical students and interns about assessments used in Faculty of Medicine, Jeddah, KSA.

## METHODS

A cross sectional study was conducted at the Faculty of Medicine, KAU, during the educational year 2012/2013. A multistage stratified random sample method was used to select senior medical students during their clinical years (4th- 6th year) and interns. Stratification took into consideration the gender and the grade.

A sample size was determined using the pre-established formula for sample size calculation:

n=(z^2^×p×q)/d^2^

A total sample to achieve a precision of ± 4% with a 95% Confidence Interval (CI) was 600.

The participants were requested to fulfill a validated, confidential, anonymous, self- administrated questionnaire. The content and face validity of the questionnaire was assessed by two experts. Evaluation of its internal-consistency reliability was done using Cronbach’s α and was found to be 87%.

The questionnaire inquired about participants’ personal information and their perceptions towards assessment methods regarding the comprehensiveness, their reflection of what is taught in the curriculum, and if the assessment methods challenging the participants more than making them just memorize. Respondents’ perceptions about the preferred type of traditional written exams (MCQs, long assay, short assay, etc.) were determined. Furthermore, the adequacy of the number of the MCQs in each exam and the number of MCQs exams per course were also inquired. Perceptions towards OSCE and OSPE were assessed. A 3-point Likert scale was used, with possible answers ranging from “disagree, neutral to agree”. Furthermore, students’ perceptions towards relatively recent non-traditional methods as log book, open-book and peer assessment were also determined.

### Ethical statement

The protocol of the study was approved by the Institutional Review Board of KAU; (Reference No: 1011-13). It conformed to the ethical standards of the Helsinki. Administrative approvals were also taken.

Data was analyzed using SPSS version 21 (SPSS Inc, Chicago, Ill., USA). Both Descriptive and analytical statistics were used. The chi-square test was used to compare between proportions. Significance was considered at *p*< 0.05.

## RESULTS

A total of 600 senior medical students and interns participated in the study with a male to female ratio of 1:1.2. It was found that 24.0%, 35.7%, 24.7% & 15.6% of participants enrolled in the fourth, fifth, sixth year & internship, respectively.

Regarding the preferred written assessment format, MCQs was the most commonly preferred exam formats by both genders, followed by the short assay. Table-I shows that females preferred MCQs slightly more (57.9%) than males (55.5%). On the other hand, a higher percentage of males (4.5%) preferred long assay compared to females (0.9%). A statistical significant difference was present between both genders regarding the preferred written assessment format (χ^2^ = 8.23, *p* < 0.05).

**Table-I:** Preferred type of written assessment methods by gender of medical studentsand interns in King Abdulaziz University.

Assessment Gender	Male		Female		Total		Fisher’s Exact Test (*p*)
	No.	%	No.	%	No.	%	
MCQs	174	55.5	194	57.9	341	56.8	8.23
Short assay	87	32.8	110	32.8	197	23.8	(< 0.05)
Long assay	12	4.5	3	0.9	15	2.6	
Others	19	7.2	28	8.4	47	7.8	
Total	265	100	335	100	600	100	

Only 39.5% of participants agreed that their assessment methods are comprehensive and a similar percentage (35.8%) agreed that assessments reflect what is taught in the curriculum. (Table-II). Furthermore, 41.2% of them agreed that assessment strategies are challenging more than making them memorize. Concerning written exams, 44.3% of respondents viewed that MCQs exams are fair format while 51.0% agreed that the number of MCQs are enough in each exam. Regarding clinical assessment, 74.1% of participants, who were already examined by OSCE (555 participants), agreed that OSCE is a good format for assessing clinical competencies, and that the numbers of OSCE stations are appropriate. On the other hand, 35.7% of them agreed that the time assigned to each station is enough. Furthermore, 70.6% of participants viewed that OSPE is a good format of clinical assessment and 66.3% agreed that the number of OSPE stations is fair. On the other hand, only 51.3% of them viewed that the time allocated for each station is enough.

**Table-II:** Degree of agreement of medical students and interns regarding different assessment methods.

Degree of agreement Assessment	Agree		Neutral		Disagree	
	No.	%	No.	%	No.	%
*General assessment methods:*					
Comprehensiveness of assessment	237	39.5	85	14.2	278	46.3
Reflection of the curriculum	215	35.8	105	17.5	280	46.7
Challenging more than memorizing	247	41.2	94	15.7	259	43.1
*Written Exams:*					
MCQs are fair assessment format	266	44.3	74	12.4	260	43.3
Number of MCQs exams are enough / exam	306	51	93	15.5	201	33.5
OSCE Exam*:					
A good method for assessing clinical competence	411	74.1	53	9.5	91	16.4
Number of stations are appropriate	371	67.1	82	14.8	100	18.1
Duration of each station is appropriate	214	35.7	50	8.3	288	48.0
OSPE Exam*:					
A good method for assessing clinical competence	415	70.6	77	13.1	96	16.3
The number of stations in OSPE is enough	390	66.3	120	20.4	78	13.3
Duration of each station is appropriate	302	51.3	102	17.3	184	31.4

* Only 555 took OSCE

* Only 588 answered question of OSPE.

Analysis of the results revealed that about one- half (49.2%) of students examined by OSCE preferred to be examined in real patients, followed by the simulated patients (36.3%), manikins (11.6%), video tapes (2.2%), while 0.7% preferred more than one type.

More proportion of males (61.3%) preferred peer assessment compared to females (50.8%), with a statistical significant difference (χ^2^ = 6.43, *p*< 0.05). Table-III. On the other hand, there are no statistical differences between both genders regarding their preferences of other non-traditional assessment methods as log books, open book exams and seminars (*p*> 0.05).

**Table-III:** Relationship between perception of medical students and interns about new methods of assessment and gender.

Assessment Gender	Male		Female		Total		χ^2^ (*p*)
	No.	%	No.	%	No.	%	
*Peer assessment:*						
Agree	155	61.3	169	50.8	324	55.3	6.43
Disagree	98	38.7	164	49.2	262	44.7	(0.01)
*Log book:*						
Agree	174	68.5	212	63.9	386	65.9	1.45
Disagree	80	31.5	120	36.1	200	34.1	(0.22)
*Open book exam:*						
Agree	148	58.3	184	55.4	332	56.7	0.48
Disagree	106	41.7	148	44.6	254	43.3	(0.49)
*Seminars:*						
Agree	101	39.8	94	28.1	195	33.3	8.58
Disagree	153	65.2	238	20.0	391	66.7	(0.003)
Total	254	34.3	332	56.7	586	100	

## DISCUSSION

The results of the current study found that only about two-fifths of participants agreed that assessment methods are comprehensive, reflect what they taught in the curriculum and challenging them more than making them memorize. This indicates that the assessment plan needs further improvement to meet the expectations of medical students.

MCQs have been widely used for summative assessment in undergraduate medical education because of their convenient standardization, efficient testing for large classes and a broad sampling of knowledge.^1^ In the current study, MCQs exams were preferred as a written assessment format more than assay which agrees with other studies.^15^-^17^

OSCE was widely accepted format for assessing clinical competence which coincides with many other studies.^18^,^19^ Furthermore, only 35.7% of respondents reported that the time assigned for each OSCE station is adequate which agrees with results of other studies.^20^,^21^ On the contrary, this disagrees with results from a study done in Riyadh’s where all students agreed that the time allocated for stations in OSCE was adequate.^19^ This discrepancy may be due to differences in sample sizes or type of exam.

Our results revealed that 70.6% of medical students agreed that OSPE is a good way of assessment of clinical competencies. This is in line with studies from India^22^ and Nepal.^23^ However, only, about one-half of our participants felt that the time allotted for each OSPE station was adequate which coincides with the results from the Indian study.^22^

Regarding new assessment methods, the current results showed that males had significantly better perception towards peer assessment compared to females. This result coincides with results of Consorti et al., 2013, who conducted an evaluation of peer physical examination among medical and osteopathic students in Italy. They found that female medical students showed a higher level of concern regarding peer assessment compared to males.^24^ Another study done in the USA found a higher proportion (80.4%) of internal medicine residences (Mayo, Rochester and Minnesota Clinics) agreed that peer evaluation is important for their professional development.^25^ The causes of higher rate from the USA compared to the current study may be because their study was conducted among residence or may be because they used to be examined by such method of assessment.

Concerning the students’ perception towards log book, our results illustrated that 68.5% of respondents agreed that it is a useful assessment format. This result concurs with the result of a survey done among UK and Irish medical students which found that log book was considered a useful way of assessment by 60% of respondents.^15^

### Strengths of the study

To the best of our knowledge, this is the first work done among a large sample of medical students and interns (both genders) in Jeddah for providing a comprehensive view about their perception towards different types of assessment.

## Conclusion

Only about two-fifths of respondents agreed that their assessment methods are comprehensive, reflect what they are taught in their curriculum and challenging them more than making them memorize. MCQs were the most commonly preferred written assessment format followed by the short essay. Regarding clinical exams, the majority of participants agreed that OSCE and OSPE are good assessment methods and that the numbers of exam stations are appropriate. Males had significantly better perception towards peer assessment compared to females. Furthermore, about two-thirds of respondents agreed that log book is a useful assessment format. Our assessments need further improvement for better preparation of medical students for their future role as physicians. Assessment should be designed prospectively along with learning outcomes. All the “outcomes for graduates” need be assessed at appropriate points during the curriculum. Adding more innovative methods of assessment as open book, self and peer assessment is required. Nowadays, our Faculty of Medicine in KAU is establishing a new Assessment Unit for this purpose. Additional studies are recommended for a better understanding of students’ perception towards different innovative assessment formats.
